# Enhanced Gas Sensitivity Characteristics of NO_2_ Sensor Based on a Silicon Micropillar Design Strategy at Room Temperature

**DOI:** 10.3390/s25206406

**Published:** 2025-10-17

**Authors:** Zhiyuan Zhang, An Ning, Jian-Jun Zhu, Yi-Yu Yue, Zhi-Qiang Fan, Sai Chen

**Affiliations:** 1 School of Integrated Circuit Science and Engineering, Beihang University, Beijing 100191, China; 2 School of Electrical and Control Engineering, North China University of Technology, Beijing 100144, China; 3 State Kay Laboratory of NBC Protection for Civilian, Beijing 102205, China; 4 School of Electronic and Information Engineering, Beihang University, Beijing 100191, China

**Keywords:** rGO/SnO_2_, gas sensor, silicon micropillar, gas-sensitive

## Abstract

In this study, two types of gas sensors—silicone-based interdigital electrode and silicon micropillar sensors based on rGO and rGO/SnO_2_—were fabricated. Their gas-sensing performance was investigated at room temperature. First, interdigital electrodes of different channel widths were fabricated to investigate the impact of the channel width parameter. Subsequently, the rGO/SnO_2_ doping ratio in the composite material was varied to identify the optimal composition for gas sensitivity. Additionally, triangular and square-arrayed silicon micropillar substrates were fabricated via photolithography and inductively coupled plasma etching. The rGO/SnO_2_-based gas sensor on a silicon micropillar substrate exhibited an ultra-high specific surface area. The triangular micropillar arrangement of rGO/SnO_2_-160 demonstrates the best performance, showing approximately 14% higher response and a 106 s reduction in response time compared with interdigital electrode sensors spray-coated with the same concentration of rGO/SnO_2_ when tested at room temperature under 250 ppm NO_2_. The optimized sensor achieves a detection limit as low as 5 ppm and maintains high responsiveness, even in conditions of 60% relative humidity (RH). Additionally, the repeatability, selectivity, and stability of the sensor were evaluated. Finally, structural and morphological characterization was conducted using XRD, SEM, TEM, and Raman spectroscopy, which confirmed the successful modification of rGO with SnO_2_.

## 1. Introduction

In response to air pollution and safety concerns, the rapid development of gas sensor technology now allows for the effective detection of gases, including acetone, ethanol, and nitrogen monoxide [1,2,3]. Nitrogen dioxide (NO_2_) is a toxic gas used in industrial production and daily applications that is harmful to the environment, causing pollution of water, soil, and the atmosphere. Furthermore, long-term exposure to low concentrations of NO_2_ can lead to poisoning. Therefore, NO_2_ gas-sensitive sensors are suitable for the real-time and accurate detection of toxic gas leaks in various environments, requiring high responsivity, stability, and repeatability.

In recent years, significant progress has been made in the development of room-temperature NO_2_ gas sensors. Metal oxides such as ZnO have attracted widespread attention due to their low cost and good stability [4]. To further improve the response speed, recovery characteristics, and responsivity of ZnO, two-dimensional materials, such as MoS_2_, have been employed for modification, leveraging their large specific surface area and high electron mobility to optimize sensor performance [5]. Moreover, novel Ag_2_Te nanowires, as an n-type semiconductor material, exhibit high responsivity and humidity resistance, even at 1 ppm NO_2_, owing to their high NO_2_ adsorption energy, low water adsorption energy, and excellent electrical conductivity [6]. The continuous development of these materials has significantly enhanced the overall performance of room-temperature NO_2_ sensors. Currently, carbon-based materials for such devices primarily focus on two categories: carbon nanotubes and graphene. Carbon nanotubes possess a high specific surface area and are easily modifiable, allowing for the selective detection of different gases through functionalization with specific groups.

Reduced graphene oxide (rGO) is an important derivative of graphene that is commonly used in the production of gas sensors. The specific functional groups, structural defects, and large specific surface area introduced during the oxidation–reduction process provide abundant active sites for the adsorption of gas molecules. Studies have shown that modifying rGO with two-dimensional materials, such as NiO, Mo_2_C, Ag, or WSe_2,_ can effectively increase its specific surface area and significantly enhance the responsivity, response speed, and recovery performance of gas sensors [7,8,9,10]. These composite materials offer more active sites for gas adsorption, resulting in significant changes in electrical properties and demonstrating excellent gas-sensing potential. SnO_2_ nanomaterials exhibit high porosity and a high surface area-to-volume ratio due to comprising mutually connected nanoparticles, which provide advantageous properties for enhancing gas sensor performance, including high responsivity, smart selectivity, and rapid response speed. By compositing SnO_2_ with rGO, sensing materials with superior performance can be constructed. For instance, using GO as a reaction substrate enables the self-assembly of flower-like hierarchical rGO-SnO_2_ nanostructures, the high surface energy of which facilitates gas adsorption. However, such sensors typically require operating temperatures between 60 and 125 °C [11,12]. To further improve the room-temperature performance and gas response characteristics, recent studies have introduced a third component into the rGO-SnO_2_ system, which effectively enhances the room-temperature sensing properties of the material [13,14].

Based on the aforementioned studies, this work introduces a three-dimensional micropillar structure into the rGO-SnO_2_ composite, significantly increasing the specific surface area and thereby enhancing its room-temperature NO_2_ sensing performance. The developed sensor exhibits a distinct response to NO_2_ at concentrations as low as 5 ppm, with a response time of 170 s at room temperature—faster than that reported in [11]—and offers excellent stability, providing a new strategy for high-performance room-temperature gas sensors.

## 2. Experimental Details

### 2.1. Silicon Interdigital Electrode Fabrication

**Figure 1:**
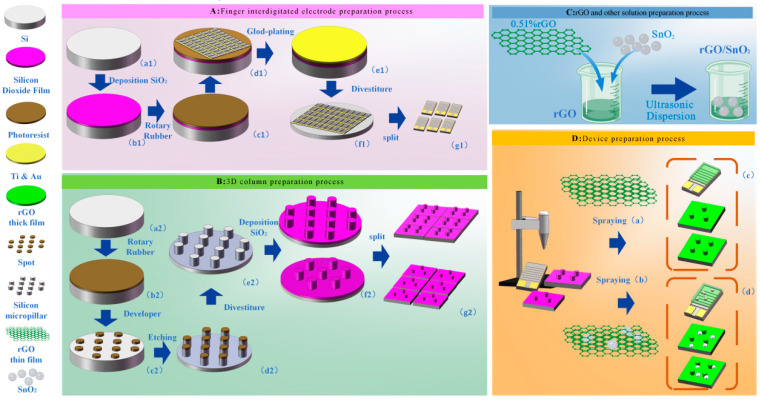
Silicon-based NO_2_ sensor preparation process flow chart.

The fabrication process of the interdigital electrode sensor, as shown in Figure 1A, was carried out step by step as follows: First, the process began with the preprocessing of an n-type (100) silicon wafer using plasma cleaning, followed by washing with acetone, drying with N_2_, and baking on a hot plate at 200 °C. Subsequently, an approximately 400 nm thick silicon dioxide insulating layer was grown via Plasma-Enhanced Chemical Vapor Deposition (PECVD) (as shown in Figure 1(A,b_1_)). Then, S-1818 photoresist was spin-coated onto the wafer at 4000 rpm for 60 s to form a uniform film (as shown in Figure 1(A,c_1_)). The wafer was pre-baked at 95 °C and then exposed to UV light for 16 s using a mask aligner. After development in a developer for 40–50 s, rinsing, and drying, the target photoresist pattern was obtained (as shown in Figure 1(A,d_1_)). Next, a double-layer metal film of 40 nm Ti and 40 nm Au was deposited under low-temperature conditions of 50 °C (as shown in Figure 1(A,e_1_)). Subsequently, lift-off processing was completed via alternating ultrasonic treatments in acetone and alcohol to remove the residual metal and photoresist, forming the interdigital electrode structure (as shown in Figure 1(A,f_1_)). Finally, a dicing saw was used to separate each interdigital electrode unit (as shown in Figure 1(A,g_1_)).

### 2.2. Silicon Micropillars Fabrication

The process for the silicon micropillar sensor is shown in Figure 1B. First, ma-N2403 photoresist was spin-coated onto a silicon wafer and then baked at 90 °C for 1 min, resulting in a final thickness of about 350 nm (as shown in Figure 1(B,b_2_)). Afterwards, a dot-array photolithography mask was drawn using L-Edit software, and Electron Beam Lithography (EBL) was used for exposure with a beam current of 1.3–1.7 nA and a dose of 100 μC/cm^2^, after which the sample was dried with N_2_ gas (as shown in Figure 1(B,c_2_)). The sample was then etched using an Inductively Coupled Plasma (ICP) etcher with the Bosch process to achieve a silicon micropillar etching depth of 10 μm (as shown in Figure 1(B,d_2_)). Following this, the sample was ultrasonically cleaned in acetone to remove the photoresist residue, washed with alcohol, and dried with N_2_ (as shown in Figure 1(B,e_2_)). Subsequently, a silicon dioxide insulating layer about 400 nm thick was grown via PECVD (as shown in Figure 1(B,f_2_)). In the final step, a knife-head slicer was used to cut the wafer into square sample pieces (as shown in Figure 1(B,g_2_)).

### 2.3. Gas Sensor Fabrication

Figure 1C shows that an rGO solution with a concentration of 0.51% was diluted 10-fold with deionized water. Next, the diluted solution was subjected to 30 min of ultrasonic treatment to ensure thorough dispersion, ultimately obtaining a uniformly textured rGO aqueous dispersion (Figure 1C, rGO). Subsequently, varying amounts of SnO_2_ were added to the rGO solution, and ultrasonic treatment was performed again to obtain a mixed solution of rGO and SnO_2_ (Figure 1C, rGO/SnO_2_). During this process, SnO_2_ uniformly adheres to the rGO surface. Following ultrasonic treatment, the rGO solution formed a finer mist during spraying, resulting in more uniform film formation and effectively preventing spray gun clogging. Next, observe Figure 1D; a total of 0.5 mL of rGO (Figure 1C) was loaded into the spray gun and sprayed onto the substrate (Figure 1(A,g_1_),(B,g_2_),(D,a)) to obtain the sensor (Figure 1(D,c)). Next, the solution was exchanged for the rGO/SnO_2_ solution (Figure 1C, rGO/SnO_2_), and the procedure was repeated to obtain the sensor (Figure 1(D,b),(D,d)). During spraying, a uniform and slow speed was maintained to prevent visible droplets of the rGO solution from forming on the substrate surface, ensuring high-quality film formation. The sprayed sensors were immediately placed in a drying oven and dried at 80 °C for approximately 120 min before testing for NO_2_ gas detection.

### 2.4. Characterization

The gas-sensing performance of the rGO/SnO_2_ sensor was evaluated under dynamic testing conditions at 30 °C and 40% RH, with chamber humidity monitored in real time. Response–recovery curves were measured for NO_2_ concentrations ranging from 50 to 250 ppm. A concentration of 250 ppm NO_2_, relevant to environmental monitoring scenarios, was emphasized for detailed assessment. The primary performance metrics investigated included the response to different gas concentrations, repeatability, and the response and recovery times (t_resp_ and t_rec_). The response time (t_resp_) and recovery time (t_rec_) are defined as the time required for the sensor resistance to reach 90% of its maximum or minimum value upon exposure to or removal of the target gas, respectively.

The synthesized materials were characterized using multiple techniques. The SnO_2_ nanoparticles employed had diameters ranging from 50 to 70 nm. Surface morphologies were examined by scanning electron microscopy (SEM, Zeiss Sigma 360, Oberkochen, Germany). The crystallinity and crystal phase were analyzed using X-ray Diffraction (XRD) with a diffractometer (Bruker Company, Coventry, UK) equipped with a high-intensity Cu Kα source (λ = 0.154 nm) and a scan rate of 13°/minute over a 2θ range of 5° to 90°. Furthermore, Raman spectroscopy was conducted with a HORIBA (Kyoto, Japan) spectrometer, and the characteristic peaks of rGO were identified from the obtained spectra.

## 3. Results and Discussion

### 3.1. The Performance of the Sensor by Optimizing Device Structure Performance

In our experiments, we primarily focused on the gas-sensing performance parameters of response, response time, and recovery time. We focused more on analyzing the gas-sensing characteristics of sensors for NO_2_ using response values rather than the response itself. Among these parameters, the response is defined as R_t_/R_0_ [11], where R_0_ is the value of resistance in N_2_ and R_t_ is the value of test resistance. The gas-sensitive detection device has a preset time of 120 s to inflate the background gas, and the response time (t_resp_) is the time it takes for the sensor to reach 90% of the steady-state resistance value after contact with the target gas. Similarly, recovery time (t_rec_) refers to the time it takes for the resistance to return to 90% R_0_ after NO_2_ is removed from the test chamber. Additionally, the responsivity–concentration gradient and response recovery time of the sensor to NO_2_ can be evaluated by setting the pulse ventilation mode in the test system. First, the background gas is continuously passed in until the resistance is stable. Then the test gas and background gas are alternately passed in for 300 s, and the concentrations of the test gas are 50, 100, 150, 200, and 250 ppm. The response and recovery time of the gas sensor to NO_2_ are tested in response and recovery modes. Similarly, the background gas needs to be passed in until the resistance is stable. Then, the test gas, with a concentration of 250 ppm, is passed in, followed by the background gas for 120 and 300 s, respectively.

**Figure 2:**
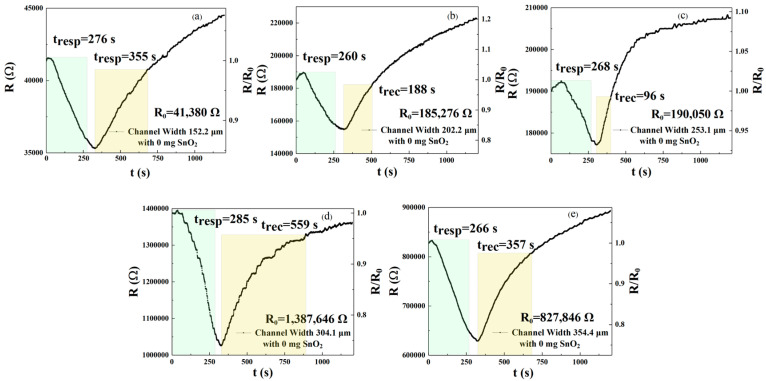
(**a**–**e**) Response and recovery curves of 250 ppm NO_2_ detected by gas sensors of different channel widths (w_1_ = 152.2 μm, w_2_ = 202.2 μm, w_3_ = 253.1 μm, w_4_ = 304.1 μm, and w_5_ = 354.4 μm) after pure rGO spraying.

In this experiment, we selected interdigital electrodes with widths of 152.2, 202.2, 253.1, 304.1, and 354.4 μm. We sprayed these with the same amount of rGO (see Figure 1C, rGO) to study the effects of interdigital electrodes of different widths on the performance of the gas sensor. Figure 2a–e show the response time and recovery time of a gas sensor detecting 250 ppm NO_2_ on five interdigital electrodes of different widths at a test temperature of 30 °C. We defined the response time and recovery time using a channel width of 304.1 μm as the reference value. The improvement rate of response time can be defined as $W_{\mathrm{resp}-w}\;$(where w represents the sample corresponding to w_1_ = 152.2 μm, w_2_ = 202.2 μm, w_3_ = 253.1 μm, w_4_ = 304.1 μm, and w_5_ = 354.4 μm):

(1) $$W_{\mathrm{resp}-w}={(w}_{\mathrm{resp}-w_{1}}-w_{\mathrm{resp}-w})/w_{\mathrm{resp}-w_{1}}\times 100\%$$

When $W_{\mathrm{resp}-w}$ is negative, it means that the response speed of this width is improved relative to the interdigital electrodes of width 304 μm, and the larger the value, the shorter the response time. Similarly, the improvement rate of the recovery time can be defined as

(2) $$W_{\mathrm{rec}-w}={(w}_{\mathrm{rec}-w_{1}}-w_{\mathrm{rec}-w})/w_{\mathrm{rec}-w_{1}}\times 100\%$$

Based on the response and recovery times of sensors of different widths (w_1_ = 152.2 μm, w_2_ = 202.2 μm, w_3_ = 253.1 μm, w_4_ = 304.1 μm, and w_5_ = 354.4 μm) (Table 1), we calculated the values for the two improvement rates for each device. The response times of the sensors (of five widths) are 276, 260, 268, 285, and 266 s, and the recovery times are 355, 188, 96, 559, and 357 s, respectively. Sensors of width w_1_, w_2_, w_3_, and w_5_ reduced the response times by 3.1%, 8.8%, 6%, and 6.7%, respectively, compared with gas-sensitive sensors of width w_4_, while recovery times for widths w_1_, w_2_, w_3_, and w_5_ decreased by 36.5%, 66.4%, 82.8%, and 36.1%, respectively. Based on these results, the most significant improvement in response speed was observed in sensors with a channel width of w_2_ = 202.2 μm, resulting in an 8.8% reduction in response time. In contrast, the best recovery speed was achieved by sensors with a channel width of w_3_ = 253.1 μm, resulting in an 82.8% reduction in recovery time.

The sensors’ responsivity to NO_2_ can be calculated from the data on the right axis in Figure 2a–e. The responsivity values of the gas sensors (of five widths) are 1.172, 1.197, 1.073, 1.352, and 1.316, respectively. As can be seen from the above data, when comparing w_1_, w_2_, w_3_, and w_5_, the responsivity values are the highest under the w_4_ condition. We define the responsivity ∆R, the slope of the response–concentration linear fit line [15], as

(3) $$∆R={(R}_{Y}-R_{X})/R_{X}\times 100\%$$

where R_Y_ is the analysis value, and R_X_ is the reference value. When ∆R is positive, it means that the responsivity of the gas sensor is increased under Y conditions relative to X, and the larger the value, the greater the increase. When ∆R is negative, it means that the responsivity of the gas sensor under condition Y is lower than that under condition X. The higher the absolute value of ∆R, the greater the decline. We selected X = w_3_ to analyze the device responsivity values for all Y values. At 250 ppm NO_2_ concentration, the ∆R of w_1_, w_2_, w_4_, and w_5_ is 9.22%, 11.56%, 25.72%, and 22.65%, respectively. Based on the above analysis, the improvement rate of the w_2_ response recovery is higher than that of other sensors’ rates, indicating that the response time is shorter, although the responsivities of w_4_ and w_5_ are higher. Therefore, gas sensors with a channel width of w_2_ have better gas detection characteristics. Next, we present a further exploration of the effects of doping with varying amounts of SnO_2_ on the gas detection performance of these devices.

**Figure 3:**
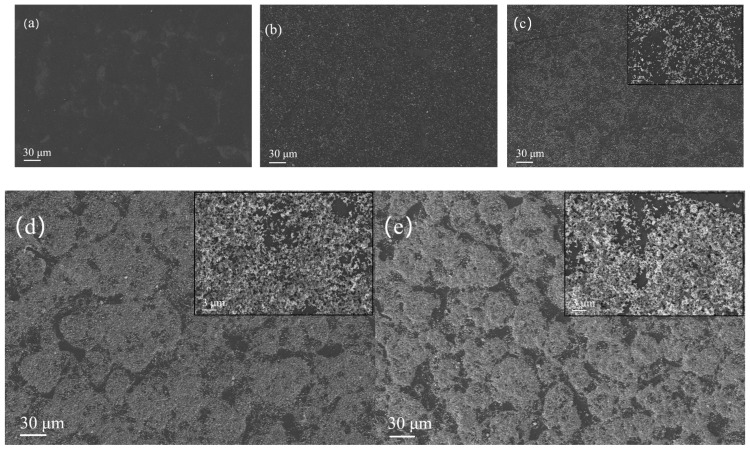
(**a**–**e**) Microstructure of rGO/SnO_2_ at different concentrations (0 mg for (**a**), 10 mg for (**b**), 20 mg for (**c**), 100 mg for (**d**), and 160 mg for (**e**)) under scanning electron microscopy after being applied to interdigital electrodes.

The image series shown in Figure 3 displays scanning electron microscopy of rGO/SnO_2_ composite gas-sensitive materials with silicon-based interdigital electrodes (width: 202.2 μm). Figure 3a–e correspond to samples with SnO_2_ doping amounts of 0, 10, 20, 100, and 160 mg, respectively. After drying at 80 °C for 2 h, the bright white area in the microstructure consists of SnO_2_ particles, and the deep black substrate is an rGO film. The upper right corners of Figure 3c–e provide a magnified view of the key area, which clearly shows how rGO/SnO_2_ particles are distributed on the surface of the gas sensor. Despite there being some incomplete coverage areas, small-scale pores do not significantly affect the charge transport performance of the gas-sensitive film, enabling effective contact between the material and the electrode; the composite layer above the sensor channel forms a stable electrical connection with the electrode, ensuring efficient carrier migration when the target gas is exposed. Additionally, the oxygen-containing functional groups on the surface of rGO may anchor SnO_2_ nanoparticles through chemical bonding, significantly enhancing the sensor’s responsivity to NO_2_.

**Figure 4:**
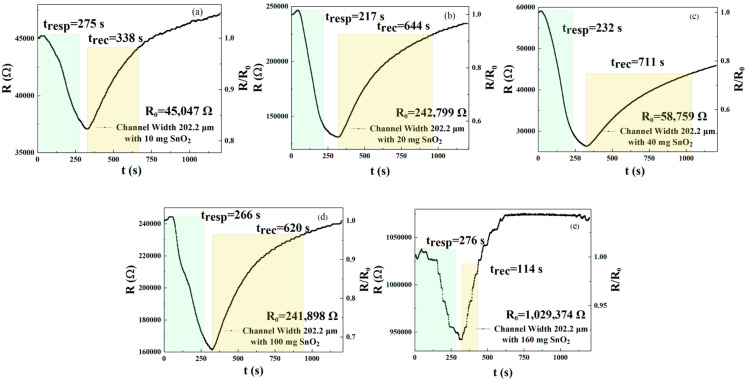
(**a**–**e**) Response and recovery curves of gas sensors sprayed with different concentrations of SnO_2_ and rGO solution (m_1_ = 10 mg, m_2_ = 20 mg, m_3_ = 40 mg, m_4_ =100 mg, and m_5_ = 160 mg), detected at 250 ppm NO_2_.

In this experiment, we prepared an rGO solution doped with five different amounts of SnO_2_ and sprayed it onto an interdigital electrode surface with the channel width that had the best performance in the previous experiment. We then used this setup as a sensor in the presence of NO_2_ at a concentration of 250 ppm. As shown in Figure 4a–e, the response recovery time and responsivity of the five sensor devices were tested for gas responsivity, respectively. Figure 4a–e and Table 2 describe the response and recovery curves and times for the 250 ppm NO_2_ condition for five different concentrations (in milligrams) of SnO_2_ in rGO solution. For these sensors, the response times for the five different concentrations (in milligrams) were 275, 217, 232, 266, and 276 s, respectively. The recovery times were 338, 644, 711, 620, and 114 s, respectively. According to Formulas (1) and (2), sensors doped with 10 mg (m_1_), 20 mg (m_2_), 40 mg (m_3_), and 100 mg (m_4_) reduced response times by 0.36%, 21.4%, 15.9%, and 3.6%, respectively, when compared with gas-sensitive sensors doped with 160 mg (m_5_). Sensors doped with 10 mg (m_1_), 20 mg (m_2_), 40 mg (m_3_), and 100 mg (m_4_) had a reduction in recovery time of −196.5%, −464.9%, −523.7%, and −443.9%, respectively. Therefore, the sensor doped with 20 mg shows the best response and recovery speed.

The response curves for NO_2_ and sensors doped with different amounts of SnO_2_ can also be calculated using Figure 4a–e. When doped with 10, 20, 40, 100, and 160 milligrams, the response values were 1.215, 1.849, 2.228, 1.499, and 1.092, respectively. Based on the above data, the sensor doped with 40 mg (m_3_) exhibits the best response when compared with the m_1_, m_2_, m_4_, and m_5_ gas-sensitive sensors, respectively. According to Formula (3), the responsivity of the m_2_ = 20 mg sensor increased by 52.1%, −260%, 23.3%, and 69.32%, when compared to the m_1_, m_3_, m_4_, and m_5_ gas-sensitive sensors, respectively. The above results show that the rGO solution doped with 20 mg of SnO_2_ has a high response value compared to the other doping amounts. Therefore, for this specific solution concentration, it is possible to adjust the width of the interdigital electrode to improve the coverage rate of the rGO film on the non-insulating layer (on the surface of the substrate), thereby more effectively adjusting the gas responsivity of the sensor device. We will discuss this in detail below.

**Figure 5:**
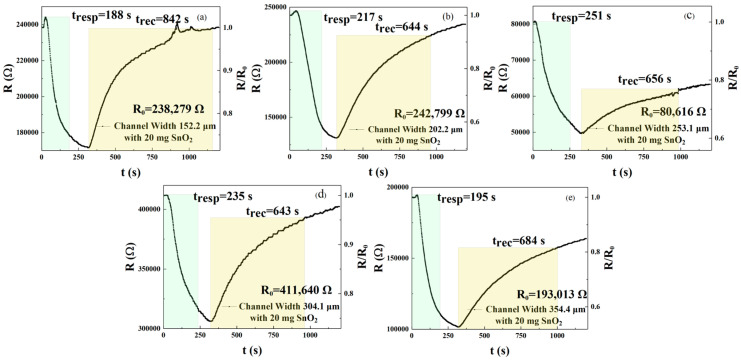
(**a**–**e**) Response and recovery detected by 250 ppm NO_2_ gas sensors of different channel widths (w_1_ = 152.2 μm, w_2_ = 202.2 μm, w_3_ = 253.1 μm, w_4_ = 304.1 μm, and w_5_ = 354.4 μm) sprayed with 20 mg SnO_2_/rGO solution.

As shown in Figure 5a–e, in this experiment, we selected the m_2_ doping concentration as the parameter for preparing the next sensor samples. Figure 5a–e and Table 3 describe the response and recovery curves and times of sensors with different interdigital electrode widths sprayed with an rGO solution doped with 20 mg of SnO_2_. The results show response times of 188, 217, 251, 235, and 195 s and recovery times of 842, 644, 656, 643, and 684 s for the five sensors, respectively. According to Formulas (1) and (2), sensors with w_1_, w_2_, w_3_, and w_5_ channel widths reduced response times by 25.10%, 13.55%, 6.37%, and 22.31%, respectively, compared with the sensor of channel width w_3_ = 253.1 μm. Sensors with w_1_, w_2_, w_3_, and w_5_ channel widths exhibit reductions in recovery time of −28.35%, 1.83%, 1.98%, and −4.27%, respectively. Therefore, sensors with a channel width of w_1_ = 152.2 μm exhibit the best response and recovery speed. When the channel width is w_1_ = 152.2 μm, w_2_ = 202.2 μm, w_3_ = 253.1 μm, w_4_ = 304.1 μm, and w_5_ = 354.4 μm, the responses were 1.388, 1.849, 1.621, 1.345, and 1.899, respectively, at the concentration of 250 ppm NO_2_. The results demonstrate that the sensor with a channel width of w_5_ = 354.4 μm exhibited the best response compared with channel widths of w_1_, w_2_, w_3_, and w_4_. According to Formula (3), a channel width of w_5_ = 354.4 μm improves responsivity by 36.82%, 2.70%, 17.15%, and 41.19%, respectively, when compared with sensors of channel width w_1_, w_2_, w_3_, and w_4_. The above results indicate that a small amount of SnO_2_ in rGO can improve performance. The reason why rGO/SnO_2_ can improve a sensor’s performance may be that the oxygen-containing functional groups on the rGO surface assist in the adsorption of SnO_2_ through chemical bonding, thereby enhancing the sensor’s responsivity to NO_2_. Second, when the added content exceeds a certain value (20 mg in this experiment), a large amount of SnO_2_ will accumulate, which can easily block the original pores of rGO and hinder the interaction between NO_2_ gas and rGO. Therefore, the 20 mg rGO/SnO_2_ condition has the best sensing performance. Gas-sensitive sensors prepared using interdigital electrodes of specific widths exhibit excellent gas responsivity characteristics at a certain concentration of NO_2_. After our analysis, the reasons may be as follows. First, when the width-to-gap ratio of the electrode changes, the influence of interface and film resistance on responsivity can be relatively reduced, thereby improving responsivity performance. Second, if the channel width is too large, it will also lead to an increase in the Schottky Barrier Height [16], which can result in higher contact resistance with the gas, causing a longer response recovery time and a decrease in responsivity. Based on the above, it is evident that increasing the rGO active site on the sensor’s surface can enhance its performance. Next, we will further explore whether the sensor’s gas detection performance can be improved by increasing the specific surface area of the sensor through the creation of three-dimensional columns.

**Figure 6:**
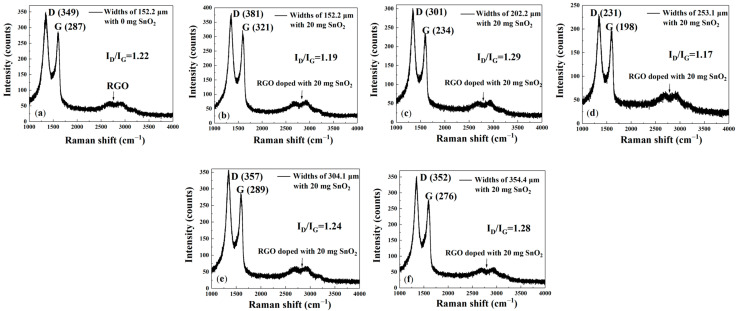
(**a**–**f**) Raman spectra of gas sensors with different channel widths (w_1_ = 152.2 μm, w_2_ = 202.2 μm, w_3_ = 253.1 μm, w_4_ = 304.1 μm, and w_5_ = 354.4 μm). Interdigital electrodes with a channel width of 202 μm, with a pure rGO coating and a coating doped with 20 mg of SnO_2_.

The Raman spectrum for pure rGO (Figure 6a) has two characteristic peaks at 1345 and 1606 cm^−1^, corresponding to the D and G peaks, respectively, confirming the presence of rGO material. The peak at 1345 cm^−1^ is the D peak, which originates from the breathing vibration mode of sp^2^ hybridized carbon atoms in the carbon lattice. Therefore, the presence of the D peak indicates structural disorder, defects, or edges in the material. The peak at 1606 cm^−1^ is the G peak, resulting from the in-plane stretching vibrations of sp^2^ hybridized carbon bonds. These two peaks are characteristic features common to all graphitized carbon materials, indicating that rGO has been successfully coated onto the interdigital electrodes. The broad peak around 2700 cm^−1^ is the 2D peak (or G’ peak), which is a second-order Raman peak arising from two-phonon resonance. Its broadened line shape and lower intensity correlate with the multi-layered and defect-rich characteristics of rGO. The clear observation of the D and G peaks in this spectrum indicates that the prepared material is a typical rGO specimen, containing both sp^2^ carbon domains and numerous defects in its structure. From Figure 6a–f, the values of I_D_/I_G_ are 1.22, 1.19, 1.29, 1.17, 1.24, and 1.28, respectively. This indicates that the reduction process successfully removed most oxygen-containing functional groups, thereby restoring the sp^2^ carbon network, and that a large number of ‘defects’ were formed at the boundaries of these small sp^2^ domains within the material. The higher defect density provides more active sites, which is beneficial for the adsorption of gas molecules.

### 3.2. Silicon Micropillar Gas Sensor Performance

**Figure 7:**
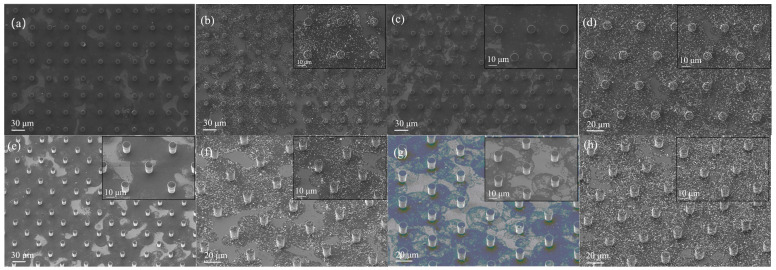
(**a**–**h**) Different rGO/SnO_2_ concentrations (0 mg for (**a**,**c**,**e**,**g**); 20 mg for (**b**,**d**,**f**,**h**)) sprayed on different surface micropillar arrangements ((**a**,**b**,**e**,**f**): square arrangement; (**c**,**d**,**g**,**h**): triangle arrangement) under scanning electron microscopy.

A series of scanning electron microscopy image systems (Figure 7) show the morphological characteristics of the rGO/SnO_2_ solution at different concentrations on the surface of the micropillars (including a pure rGO solution and a solution doped with 20 mg SnO_2_) with different spatial arrangements: a–b correspond to the square array arrangement, and c–d correspond to the triangle array arrangement. The upper right corner of each image is embedded with a magnified view of the key area, and e–h represent the oblique viewing angle of a–d, respectively. As shown in Figure 7, the three-dimensional cylinder structure exhibits significant advantages over the flat substrate. The gas-sensitive material forms an extended interface on the three-dimensional surface, greatly increasing the effective contact area with the target gas; this enables the sensor to have a faster response speed and higher detection responsivity to the target gas. 

**Figure 8:**
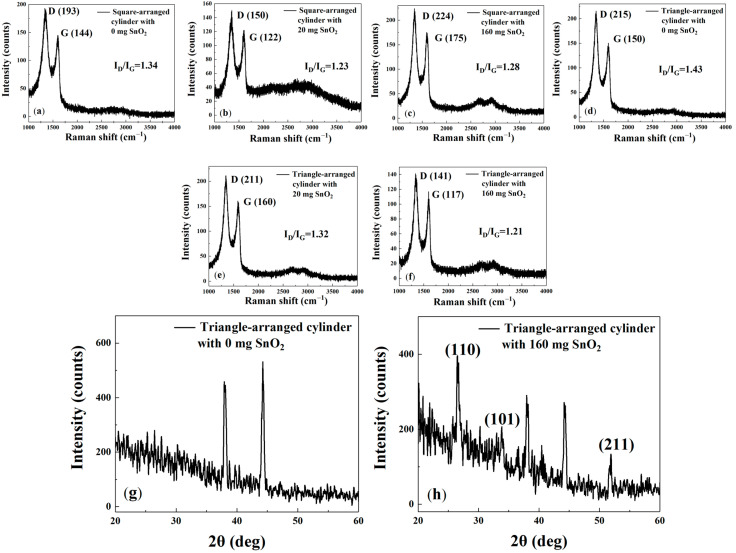
(**a**–**f**) Raman spectra of gas sensors with micropillars arranged in squares and triangles, sprayed with different amounts (milligrams) of SnO_2_ (0, 20, and 160 mg) in rGO solution. (**g**,**h**) XRD spectra of gas sensors with micropillars sprayed with different amounts (milligrams) of SnO_2_ in rGO solution.

Figure 8a–f shows that the cylindrical gas-sensitive materials exhibit a similar Raman spectrum and peaks to the interdigital electrode gas-sensitive materials, with peaks at approximately 1350, 1600, and 2700 cm^−1^. This indicates that they share similar physical properties with the interdigital electrode gas-sensitive materials. From Figure 8a–f, the values of I_D_/I_G_ are 1.34, 1.23, 1.28, 1.43, 1.32, and 1.21, respectively. These results confirm that the incorporation of SnO_2_ nanoparticles effectively disrupts the rGO lattice and increases defect density. Furthermore, the sensors with optimal gas response—the square array with 20 mg SnO_2_ and the triangular array with 160 mg SnO_2_—exhibit lower I_D_/I_G_ ratios, confirming that an increase in defects contributes to enhanced gas response performance.

The g and h compares the XRD spectra of rGO solutions with 160 mg SnO_2_ and without SnO_2_ doping, sprayed onto cylindrical gas-sensitive materials arranged in a triangle pattern. It can be seen that, compared with g the spectrum in h displays three main characteristic peaks, corresponding to crystal orientations: (110), (101), and (211). These three crystal orientations indicate the presence of SnO_2_ particles. The characteristic peaks can be indexed according to the standard tetragonal rutile structure (JCPDS card, no. 41-1445).

**Figure 9:**
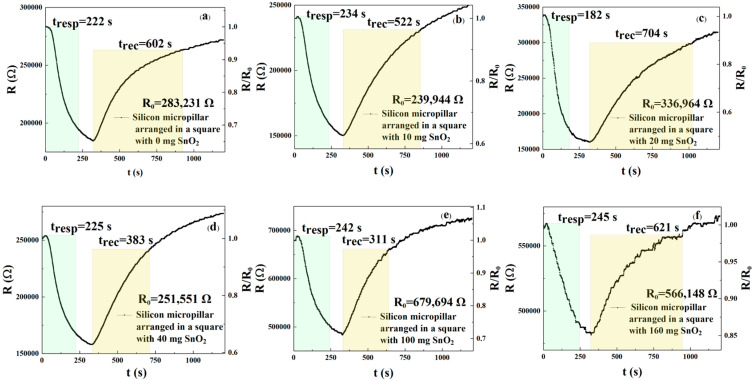
(**a**–**f**) Response and recovery curves of gas sensors with a square arrangement of micropillars sprayed with rGO solutions doped with different concentrations of SnO_2_ (m_1_ = 0 mg, m_2_ = 10 mg, m_3_ = 20 mg, m_4_ = 40 mg, m_5_ = 100 mg, and m_6_ = 160 mg) detected by 250 ppm NO_2_.

In this experiment, we selected three-dimensional cylinders made of silicon as the experimental material, which have a diameter of 6.0 μm and a distribution period of 14.5 μm. The array has a square arrangement, sprayed with six different concentrations of rGO/SnO_2_; the prepared sensors were tested for response recovery time and responsivity. Figure 9a–f and Table 4 show the response and recovery curves and times of the sensors (square arrangement; three-dimensional column) to 250 ppm NO_2_ when sprayed with six different concentrations of rGO/SnO_2_. The results show that the response times regarding the six different concentrations are 222, 234, 182, 225, 242, and 245 s, respectively, with recovery times of 602, 522, 704, 383, 311, and 621 s, respectively. According to Formulas (1) and (2), the response times of the m_1_, m_2_, m_3_, m_4_, and m_5_ sensors were reduced by 9.39%, 4.49%, 25.71%, 8.16%, and 1.22%, respectively, when compared with the m_6_ gas sensor, while the recovery times of the m_1_, m_2_, m_3_, m_4_, and m_5_ sensors were reduced by 3.06%, 15.94%, −13.37%, 38.33%, and 49.92%, respectively. Therefore, the sensor doped with 20 mg shows the best response and recovery speed. The NO_2_ response of these six sensors can be calculated from Figure 9a–f and Formula (3). When the rGO/SnO_2_ concentrations were m_1_, m_2_, m_4_, m_5_, and m_6_, the responsivity values were 1.533, 1.596, 2.101, 1.591, 1.406, and 1.175, respectively. The results show that the sensor with an rGO/SnO_2_ concentration of m_3_ = 20 mg exhibited the best responsivity compared with m_1_, m_2_, m_4_, m_5_, and m_6_, respectively. According to Formula (3), the responsivity of the sensor with m_3_ = 20 mg improved by 37.05%, 31.64%, 32.06%, 49.43%, and 78.81%, respectively, when compared to the m_1_, m_2_, m_4_, m_5_, and m_6_ sensors. For square-arranged silicon micropillar sensor surfaces, the m_3_ (20 mg) sensors perform best. Next, we investigate whether triangularly arranged silicon micropillars also impact gas detection performance.

**Figure 10:**
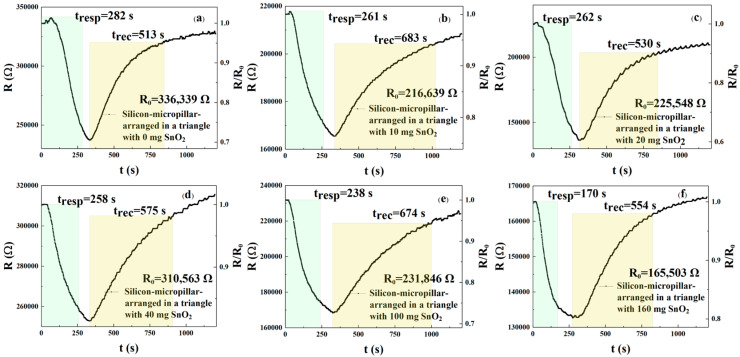
(**a**–**f**) Response and recovery curves of gas sensors with a triangular arrangement of micropillars sprayed with different concentrations of rGO/SnO_2_ solution (m_1_ = 0 mg, m_2_ = 10 mg, m_3_ = 20 mg, m_4_ = 40 mg, m_5_ = 100 mg, and m_6_ = 160 mg) detected by 250 ppm NO_2_.

In this experiment, we selected a triangular arrangement of micropillars with the same period and diameter as the square arrangement: 14.5 and 6.0 μm, respectively. These were sprayed with different concentrations of rGO/SnO_2_ solution. Figure 10a–f show the recovery curves of the sensors’ response to 250 ppm NO_2_. Figure 10a–f and Table 5 describe the response and recovery curves and times of the described sensors. The results show response times of 282, 261, 262, 258, 238, and 170 s, respectively, and recovery times of 513, 683, 530, 575, 674, and 554 s, respectively, for the six different solution concentrations. According to Formulas (1) and (2), the response time of the m_2_, m_3_, m_4_, m_5_, and m_6_ sensors was reduced by 7.45%, 7.09%, 8.51%, 15.60%, and 39.72%, respectively, when compared with the m_1_ sensor. The recovery times of the m_2_, m_3_, m_4_, m_5_, and m_6_ sensors were reduced by −33.14%, −3.31%, −12.09%, −31.38%, and −7.99%, respectively. These values indicate that for the triangular arrangement of micropillars, the m_6_ = 160 mg sensor shows the best response and recovery speed. The NO_2_ response for these six sensors can be calculated from Figure 10a–f. When the rGO/SnO_2_ concentrations were m1, m2, m3, m4, m5, and m6, the response values were 1.417, 1.308, 1.652, 1.228, 1.377, and 1.247, respectively. Based on the above data, sensors with rGO/SnO_2_ doping concentrations of m_3_ = 20 mg exhibit the best response compared with the m_1_, m_2_, m_4_, m_5_, and m_6_ sensors, respectively. According to Formula (3), the responsivity of the m_3_ = 20 mg sensor increased by 16.58%, 26.30%, 34.53%, 19.97%, and 32.48%, respectively, compared with the m_1_, m_2_, m_4_, m_5_, and m_6_ gas sensors. It is evident that for a triangular arrangement of micropillars, the 160 mg rGO/SnO_2_ concentration (m_6_) exhibits the best performance. From the above experimental results, it is clear that the response recovery time of the triangular-arrangement sensor is shorter than that of the square-arrangement sensor. The possible reasons for this are as follows. The triangular arrangement (hexagonal, close-packed structure) has a higher packing density and a more uniform gap distribution than the square arrangement. Therefore, the sensor with a triangular arrangement of silicon micropillars has a larger effective specific surface area, and the formed rGO coating may form a more continuous network, providing more active sites for interaction with NO_2_ molecules [17]. Another possibility is that the triangular arrangement may promote gas diffusion by optimizing gas permeation, enabling the rapid and uniform distribution of gas between the micropillars. This is because the triangular arrangement reduces the tail effect and equalizes the usable concentration downstream of the array, thereby improving the signal and accelerating the rate to a steady state, with a higher response and faster stability [18].

**Figure 11:**
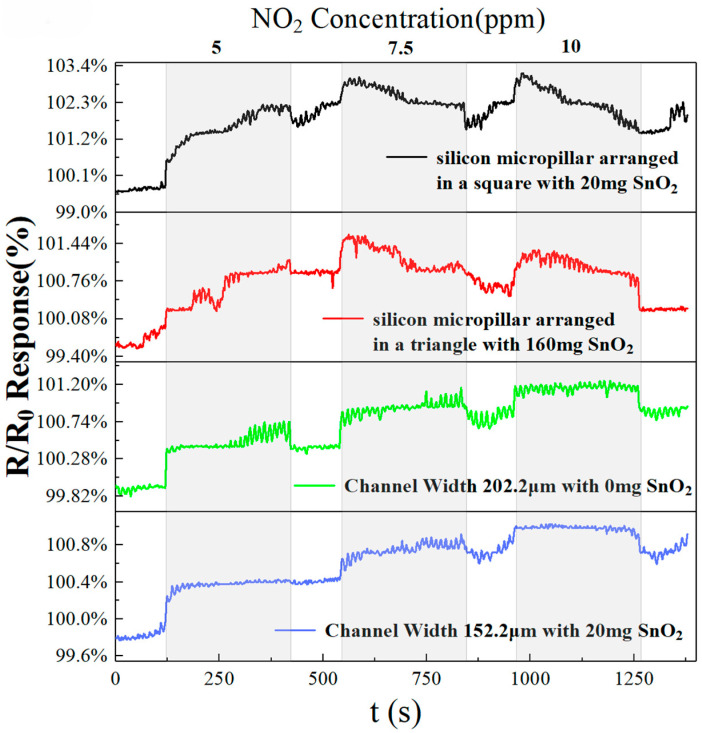
Response of various gas sensors: 160 mg doping and triangle-arranged micropillars, 20 mg doping and square-arranged micropillars, 0 mg doping and a width of 202.2 μm, and 20 mg doping and a width of 152.2 μm.

At 40 °C, we selected the best pure rGO sensor, SnO_2_-doped sensor, triangle-arranged micropillars sensor, square-arranged micropillars sensor, and interdigital electrode sensor, with the test curve for NO_2_ gas shown in Figure 11. The sensors have similar response and recovery times over a NO_2_ concentration range of 5 to 10 ppm. The four optimized sensors all respond to NO_2_ gas concentrations of 5, 7.5, and 10 ppm, indicating that the fabricated sensors can also respond at low concentrations. Compared with the other sensors, the pure rGO sensor with a channel width of 202.2 µm exhibits the highest responsivity and shows a distinct recovery curve at 5, 7.5, and 10 ppm. This indicates that at room temperature, the pure rGO sensor with a channel width of 202.2 µm can achieve good response recovery performance for low concentrations of gas.

**Figure 12:**
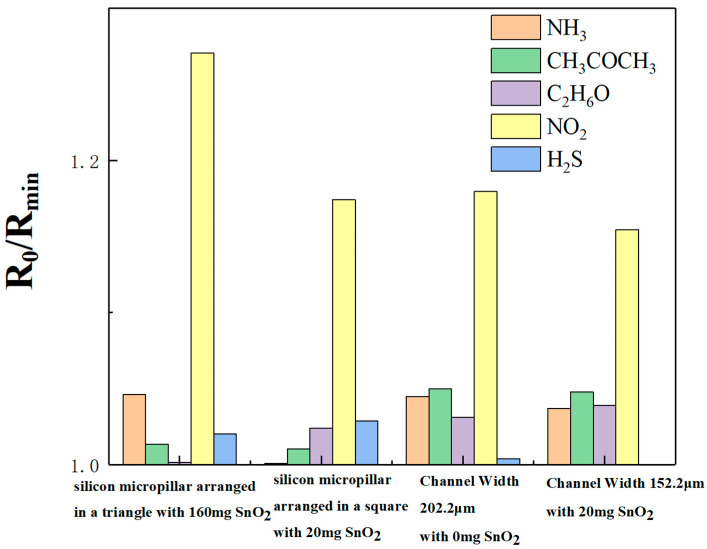
Comparison of the responsivity of a 160 mg-doped triangle-arranged micropillars sensor, a 20 mg-doped square-arranged micropillars sensor, a 0 mg-doped and 202.2 μm-width sensor, and a 20 mg-doped and 152.2 μm-width sensor to NH_3_, CH_3_COCH_3_, C_2_H_6_O, NO_2_, and H_2_S at a concentration of 50 ppm.

Additionally, the four sensors were tested for gas selectivity, as illustrated in Figure 12. All the different gases were at a concentration of 50 ppm. Responsivities to NH_3_, CH_3_COCH_3_, C_2_H_6_O, NO_2_, and H_2_S for the 160 mg-doped triangle-arranged micropillars sensor were 1.04604, 1.0133, 1.00173, 1.27071, and 1.02014, respectively. According to Formula (3), responsivity to NO_2_ increased by 21.48%, 25.40%, 26.85%, and 24.56%, respectively, when compared with NH_3_, CH_3_COCH_3_, C_2_H_6_O, and H_2_S. For the 20 mg-doped square-arranged micropillars sensor, the responsivities were 1.00082, 1.0103, 1.02413, 1.17417, and 1.02891, respectively. According to Formula (3), responsivity to NO_2_ increased by 17.32%, 16.22%, 14.65%, and 14.12%, respectively, when compared with NH_3_, CH_3_COCH_3_, C_2_H_6_O, and H_2_S. Responsivities to NH_3_, CH_3_COCH_3_, C_2_H_6_O, and H_2_S for the 0 mg-doped with a width of 202.2 μm sensor were 1.0448, 1.04997, 1.03134, 1.17961, and 1.00408, respectively. According to Formula (3), responsivity to NO_2_ increased by 12.90%, 12.35%, 14.38%, and 17.48%, respectively, when compared with NH_3_, CH_3_COCH_3_, C_2_H_6_O, and H_2_S, respectively. Finally, for the 20 mg-doped with a width of 152.2 μm sensor, the responsivities were 1.0371, 1.04789, 1.03909, 1.1545, and 0, respectively. According to Formula (3), responsivity to NO_2_ increased by 11.32%, 10.17%, and 11.11%, respectively, when compared with NH_3_, CH_3_COCH_3_, and C_2_H_6_O,. Based on the above data, when compared with NH_3_, CH_3_COCH_3_, C_2_H_6_O, and H_2_S, the sensors respond significantly more to NO_2_ gas, indicating the high selectivity of our sensors for NO_2_ gas.

**Figure 13:**
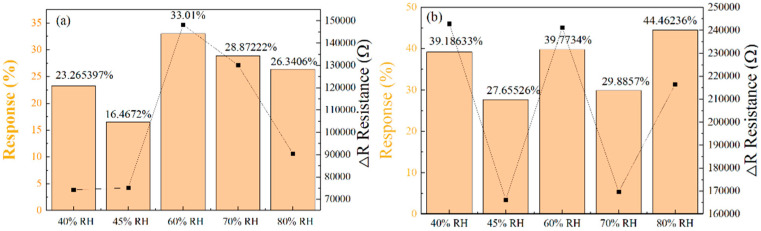
(**a**) The response variation in the gas sensors with a triangular array of micropillars to NO_2_ under different RH conditions. (**b**) The response variation in the gas sensors with a square array of micropillars to NO_2_ under different RH conditions.

To explore sensor response under different humidity conditions, we subjected the two optimized micropillar sensors to relative humidity environments of 40%, 45%, 60%, 70%, and 80%. Figure 13a,b show the relative response values of the two sensor types under varying humidity levels, corresponding to the triangular and square arrangements of micropillars, respectively. The sensors’ response to NO_2_ was significantly influenced by ambient humidity, exhibiting an initial increase followed by a decrease. At moderate humidity levels, the hydroxyl groups formed by the dissociation of adsorbed surface water acted as proton conductors, promoting NO_2_ charge transfer reactions. However, in high-humidity environments, excess water molecules form a physical barrier, preventing NO_2_ gas molecules from approaching and adsorbing onto the most critical, highly active sites, resulting in reduced responsivity. A relative humidity of 60% represents the optimal equilibrium point. At this level, the promotional effect of water molecules reaches its maximum, whereby their shielding of active sites has not yet become a significant issue, resulting in the highest response value.

**Figure 14:**
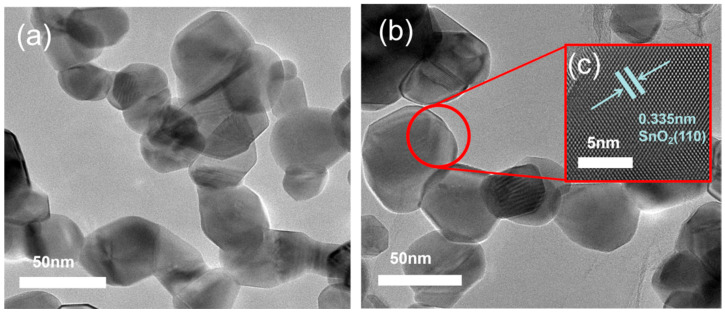
(**a**) TEM images of SnO_2_; (**b**) TEM images of rGO/SnO_2_-160; (**c**) HRTEM images of rGO/SnO_2_-160.

**Figure 15:**
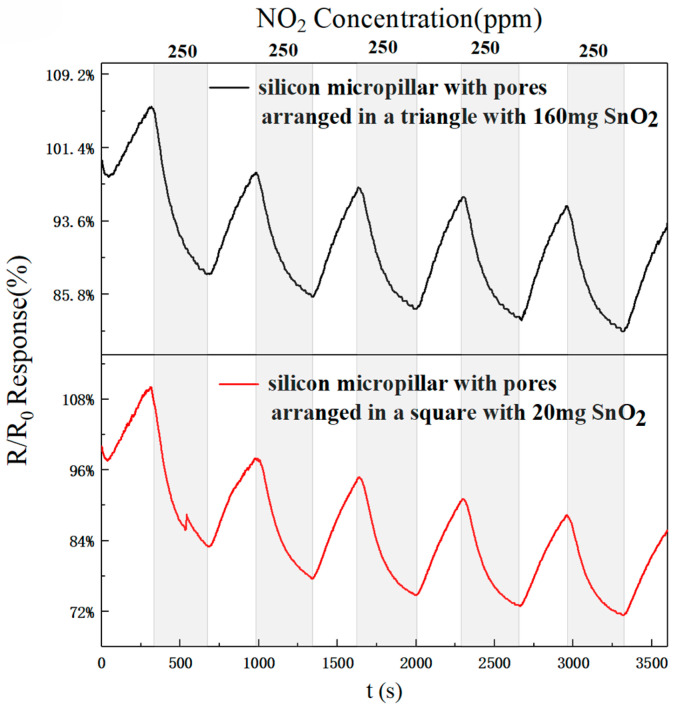
Repeatability test of gas sensor responsivity to NO_2_ response at a concentration of 250 ppm for a 160 mg-doped triangle micropillar array sensor and a 20 mg-doped square micropillar array sensor.

**Figure 16:**
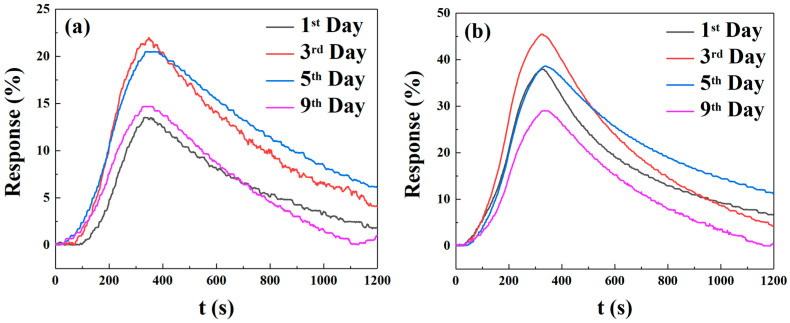
(**a**) Response and recovery times of the 160 mg-doped triangular micropillar array sensor measured on the 1st, 3rd, 5th, and 9th days. (**b**) Response and recovery times of the 20 mg-doped square micropillar array sensor measured on the 1st, 3rd, 5th, and 9th days.

It is interesting to note that in humid environments, the response values of sensors arranged in a square arrangement are higher than those of sensors arranged in a triangular arrangement. The sensors with micropillars in a triangular arrangement showed response values of 23.27%, 16.47%, 33.01%, 28.87%, and 26.34% for 250 ppm NO_2_ gas at relative humidities of 40%, 45%, 60%, 70%, and 80%, respectively. In contrast, the sensors with micropillars in a square arrangement demonstrated superior performance, with response values of 39.19%, 27.66%, 39.77%, 29.89%, and 44.46%. Figure 14a presents a TEM image of pure SnO_2_, characterized by the aggregation of numerous small nanoparticles in the dark regions. In contrast, Figure 14b shows a similar morphology, indicating a uniform dispersion of SnO_2_ nanoparticles on the rGO sheets. Furthermore, the HRTEM image in Figure 14c reveals well-defined lattice fringes with an interplanar spacing of 0.335 nm, corresponding to the (110) crystal plane of SnO_2_, providing further evidence of successful composite formation. Figure 15 shows the results of five cycles of 250 ppm NO_2_ for the more responsive sensors (triangular and square micropillar arrays). The response value remains relatively unchanged, indicating that the sensors exhibit good repeatability. To ensure the temporal stability of the test results, we selected four time points: the first, third, fifth, and ninth days. We conducted response tests for two micropillar array types of gas sensors with a target gas concentration of 250 ppm NO_2_, as shown in Figure 16. We define the “response value” as follows:

(4) $$\mathrm{Response}(R)\;=\;\left|\frac{R_{g}-R_{a}}{R_{a}}\right|$$

R_g_ is the current resistance, and R_a_ is the initial resistance [5]. The highest response values of the triangular micropillar array gas sensors at the four time points are 13.531%, 22.004%, 20.495%, and 14.700%, respectively. The response on the third day is higher than that on the first, fifth, and ninth days by 62.62%, 7.36%, and 49.69%, respectively. The highest response values of the square micropillar array gas sensors at the four time points are 37.975%, 45.447%, 38.628%, and 29.076%, respectively. The response on the third day is higher than that on the first, fifth, and ninth days by 19.68%, 17.65%, and 56.30%, respectively. From the above results, it is obvious that both types of gas sensors reached their highest response values on the third day. The optimized sensors in this work demonstrate substantially superior performance to the value of 13.27 reported in [12] for 300 ppm NO_2_, even at a lower detection concentration of 250 ppm. The responses of both optimized sensors exceeded the value reported in the reference. Notably, the 20 mg-doped square micropillar array sensor achieved optimal response values ranging from 29.17 to 45.47 over different testing days, confirming a significant enhancement in response.

We define the “limit of detection” as follows:

(5) $$\mathrm{LOD}=\frac{3\mathrm{RMS}}{k}$$

**Figure 17:**
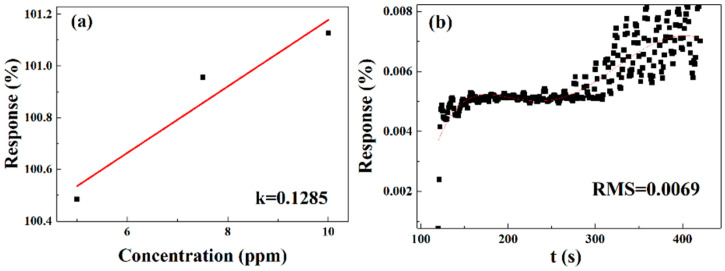
(**a**) Linear fit curve of the sensor response versus NO_2_ concentration of 0 mg-doped and 202.2 μm-width gas sensor. (**b**) Fifth polynomial fitting curve for baseline data of 0 mg-doped and 202.2 μm-width gas sensor.

Slope k can be obtained by linear fitting (Figure 17a), and the root mean square (RMS) can be calculated using the fifth polynomial linear fitting analysis of the baseline data of the 0 mg-doped and 202.2 μm-width gas sensor (Figure 17b). Finally, the theoretical LOD of the gas sensor was calculated to be 0.161089 ppm [19].

### 3.3. Gas-Sensing Mechanism

**Figure 18:**
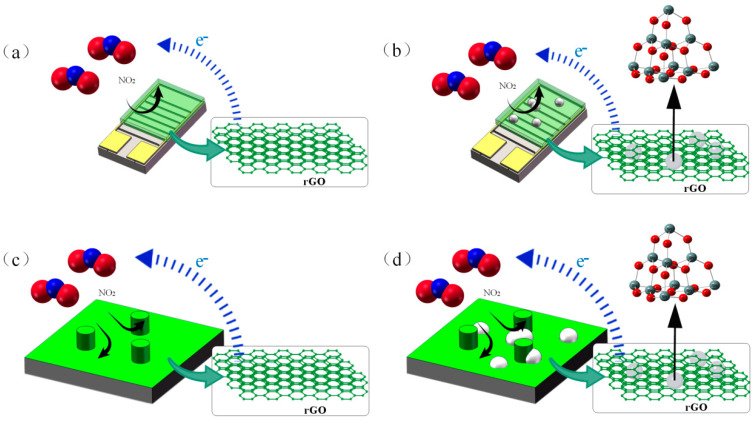
(**a**–**d**) The pathways of pure dilution of rGO and rGO/SnO_2_ spraying on interdigital electrodes and columns, respectively.

In this article, we selected rGO and composite solutions as gas-sensitive materials. The gas-spraying method enables the uniform deposition of these materials onto the sensor surface by atomizing aqueous solutions into a mist under high-pressure gas flow [20]. Figure 18 presents the microscopic behavior of the interdigital electrodes and silicon micropillar sensors sprayed with rGO and rGO/SnO_2_ solutions upon exposure to NO_2_ gas molecules. Figure 18a illustrates when NO_2_ gas contacts the sensor and is adsorbed onto the rGO surface. rGO inherently possesses a defective structure that is favorable for target gas adsorption and exhibits typical p-type semiconductor behavior. As a strong electron acceptor, NO_2_ extracts electrons from rGO upon adsorption, resulting in a charge transfer. This process increases the hole carrier density in rGO, significantly enhancing its conductivity and reducing its resistance [21].

Due to their large specific surface area, nanoparticles can easily adsorb gas molecules, thereby enhancing gas-sensing performance effectively. For instance, the modification of WO_3_ and CeO_2_ nanoparticles has been demonstrated to exhibit such effects [22,23]. This study used defective SnO_2_ nanoparticles firmly embedded within an rGO substrate to achieve tight integration (Figure 6). This configuration generates abundant active sites and produces synergistic effects, significantly enhancing the adsorption capacity for NO_2_ gas. SnO_2_ nanoparticle doping increased the surface roughness of the sensing membrane of the gas-sensing device, and the surface defects also significantly increased, collectively promoting an increase in the density of the NO_2_ adsorption active sites. By optimizing the amount of SnO_2_ nanoparticle doping, an rGO/SnO_2_ composite membrane with optimal surface morphology and defect structure was obtained. This composite material effectively enhances electron transfer capability [24]. Compared with pure rGO films, the rGO/SnO_2_ composite film possessed more active adsorption sites, facilitating greater electron transfer to the NO_2_ molecules. This process substantially increases the hole concentration within the rGO conductive layer, inducing more pronounced resistance changes and enhancing the responsivity of the device [5]. The microstructure of the corresponding sensor is shown in Figure 18b.

In this article, we employed MEMS technology to fabricate a silicon micropillar array substrate for the sensors. By atomizing and spraying aqueous solutions of rGO and rGO/SnO_2_ gas-sensitive materials using high-pressure gas, these materials were uniformly deposited onto silicon micropillar surfaces to form rGO/SnO_2_ composite films. Two silicon micropillar arrangements, namely, square and triangular arrays, were designed as the device substrates. As illustrated in Figure 18c,d, taking the triangular array as an example, the flow path of the NO_2_ gas across the sensor surface and the resulting electron transfer process are shown. This silicon micropillar array structure significantly increased the specific surface area of the rGO/SnO_2_ composite film. This enhanced the surface roughness of the film layer and introduced more surface defects, thereby substantially increasing the number of NO_2_ adsorption active sites. The synergistic effect of these microstructures effectively enhances electron transfer efficiency during the gas-sensing response process, ultimately improving the detection responsivity and overall performance of the sensor for NO_2_ gas.

## 4. Conclusions

In this study, we produced rGO/SnO_2_ sensors by increasing the number of active sites and using silicon micropillars. We experimentally prepared sensors with interdigital electrodes of varying widths, sprayed with rGO/SnO_2_ at different concentrations, as well as silicon micropillar sensors, and measured their performance under various concentrations of NO_2_ (50, 100, 150, 200, and 250 ppm). First, by varying the channel width of the interdigital electrodes, we obtained sensors with excellent gas-sensing performance. The experimental results showed that when diluted rGO was sprayed onto the surface, the sensor performed best with a channel width of 202.2 μm for the interdigital electrodes. When the NO_2_ concentration was 250 ppm, the response and recovery times of this sensor type were 260 and 188 s, respectively. We then modified the sensor by adding SnO_2_ nanoparticles to the diluted rGO solution. The results indicated that the responsivity of the sensor improved most significantly after 20 mg doping, reaching 1.849, suggesting that the addition of SnO_2_ to the sensor surface enhanced its performance. Subsequently, we determined the optimal concentration of rGO/SnO_2_ as a reference and further modified the width of the interdigital electrodes. Through experimental analysis, it was found that after doping the rGO solution with 20 mg of SnO_2_, the response time of the sensor with a finger interdigital electrode width of 152.2 μm reached 188 s, and the responsivity also significantly improved, indicating that the width modification of the finger interdigital electrodes can enhance the performance of the sensor. Finally, we utilized a 3D structure to fabricate a silicon micropillar-type surface modification gas sensor. The experimental results showed that after micropillar processing was added, the response and recovery times, as well as the responsivity of the sensor, were improved. Among these, the triangular arrangement of silicon micropillars achieved a response time of 170 s. Additionally, the responsivity of the square-arrangement sensor increased to 2.101, representing a 10.6% increase compared with the previous state, clearly demonstrating that the addition of micropillar processing can significantly enhance sensor performance. The study found that as surface modification methods, both the addition of SnO_2_ nano-treatment and the use of silicon micropillar treatment had a significant impact on improving the sensor’s response and recovery time, as well as its responsivity.

## Figures and Tables

**Table 1:** Response and recovery times of gas sensors of different channel widths after being sprayed with rGO solution.

Channel Widths (μm)	Response Time (s)	Recovery Time (s)
w_1_/152.2	276	355
w_2_/202.2	260	188
w_3_/253.1	268	96
w_4_/304.1	285	559
w_5_/354.4	266	357

**Table 2:** Response and recovery times of gas sensors with an interdigital electrode channel width of 202.2 μm sprayed with different concentrations of rGO solution.

The rGO/SnO_2_ (mg)	Response Time (s)	Recovery Time (s)
m_1_/10	275	338
m_2_/20	217	644
m_3_/40	232	711
m_4_/100	266	620
m_5_/160	276	114

**Table 3:** Response and recovery times of gas sensors with different interdigital electrode widths sprayed with 20 mg SnO_2_/rGO solution.

Channel Widths (μm)	Response Time (s)	Recovery Time (s)
w_1_/152.2	188	842
w_2_/202.2	217	644
w_3_/253.1	251	656
w_4_/304.1	235	643
w_5_/354.4	195	684

**Table 4:** Response and recovery time of gas sensors with a square arrangement of micropillars sprayed with rGO solutions doped with different concentrations of SnO_2_.

The rGO/SnO_2_ (mg)	Response Time (s)	Recovery Time (s)
m_1_/0	222	602
m_2_/10	234	522
m_3_/20	182	704
m_4_/40	225	383
m_5_/100	242	311
m_6_/160	245	621

**Table 5:** Response and recovery times of gas sensors with a triangular arrangement of micropillars sprayed with different concentrations of rGO/SnO_2_ solution.

The rGO/SnO_2_ (mg)	Response Time (s)	Recovery Time (s)
m_1_/0	282	513
m_2_/10	261	683
m_3_/20	262	530
m_4_/40	258	575
m_5_/100	238	674
m_6_/160	170	554

## Data Availability

The original contributions presented in this study are included in the article. Further inquiries can be directed to the corresponding authors.
